# Lipid Nanoparticles: Formulation, Production Methods and Characterization Protocols

**DOI:** 10.3390/foods14060973

**Published:** 2025-03-12

**Authors:** Fernanda L. Lüdtke, Thaís Jordânia Silva, Mayanny Gomes da Silva, Juliana Campos Hashimoto, Ana Paula B. Ribeiro

**Affiliations:** 1 CEB—Centre of Biological Engineering, University of Minho, 4710-057 Braga, Portugal; fludtke@ceb.uminho.pt; 2 LABBELS—Associate Laboratory, 4800-122 Guimarães, Portugal; 3 Center for Natural Sciences, Federal University of São Carlos (UFSCar), Campus Lagoa do Sino, Buri 13565-905, SP, Brazil; thais.jordania12@gmail.com; 4 Department of Food Engineering and Technology, Faculty of Food Engineering, University of Campinas, Campinas 13083-862, SP, Brazil; mayannygomes@hotmail.com (M.G.d.S.); jucampos@unicamp.br (J.C.H.)

**Keywords:** solid lipid nanoparticles, nanostructured lipid carriers, bioactive compounds, lipid crystallization, physical stability, bioaccessibility

## Abstract

Lipid nanoparticles (LNs) have emerged as advanced lipid-based delivery systems, offering an effective approach for encapsulating and protecting lipid-soluble bioactive compounds, increasing their bioavailability. Solid Lipid Nanoparticles (SLNs) and Nanostructured Lipid Carriers (NLCs) are particularly promising for bioactive compound entrapment. However, to fully exploit their potential, it is crucial to carefully select the appropriate lipid matrices and emulsifiers. This review offers a comprehensive, up-to-date examination, considering studies published in the last 15 years, of the chemical, physical, and structural characteristics of lipids employed in LN production, focusing on the key components of the formulations: lipid matrices, emulsifiers, and bioactive compounds. In addition, it provides an in-depth analysis of production methods, drawing on insights from the latest scientific literature, and emphasizes the most important characterization techniques for LNs. Key parameters, including particle size (PS), zeta potential (ZP), crystallinity, thermal behavior, morphology, entrapment efficiency (EE), load capacity (LC), and physical stability, are discussed. Ultimately, this review aims to identify critical factors for the successful production of stable LNs that efficiently encapsulate and deliver bioactive compounds, highlighting their significant potential for applications in food systems.

## 1. Introduction

Nanotechnology is defined as the application of scientific and engineering principles to the design, development, and utilization of materials with nanometric dimensions. It involves the creation and manipulation of both organic and inorganic substances at the nanoscale. Materials developed via nanotechnology display physical and chemical properties that significantly differ from those of bulk materials with identical composition [1]. Since the publication of the first study on nanotechnology in food by Moraru and colleagues in 2003, research has focused on its potential applications in food safety, functional foods, the nanoscale properties of food materials, and the development of systems for delivering bioactive compounds [2,3]. Advancements in nanotechnology research have demonstrated that nanoscale delivery systems provide superior functionality over conventional microencapsulation methods, thereby contributing to the increasing interest in nano-carrier technologies. These systems, including lipid nanoparticles (LNs), are distinguished by their high kinetic and thermodynamic stability [4]. Specifically, enriching foods with functional or bioactive compounds poses challenges related to stability under processing conditions, challenges that can be effectively addressed through nanotechnology approaches [3].

LNs have emerged as a prominent nano-sized lipid-based delivery system for encapsulating and protecting lipid-soluble bioactive compounds. They offer several advantages, including improved solubility within the food matrix, protection from environmental factors that may cause degradation (such as light, temperature, and temperature fluctuations), enhanced stability, controlled and targeted release, and increased bioavailability of bioactive compounds [5,6,7,8]. Among lipid-based delivery systems, Solid Lipid Nanoparticles (SLNs) and Nanostructured Lipid Carriers (NLCs) demonstrate considerable potential for encapsulating bioactive compounds. However, to fully realize the advantages of SLNs and NLCs, it is essential to carefully choose both the type and proportion of lipid matrices and emulsifiers in their formulation [8,9].

Structurally, SLNs are composed of lipids with a higher melting point (solid lipids), which, upon crystallization, form more ordered, tightly packed structures. This may result in less space for the entrapment of bioactive compounds. Therefore, when selecting the lipid material for the composition of SLNs, it is crucial to consider the solubility of the compound in the lipid phase. NLCs, on the other hand, are composed of lipids with both higher and lower melting points (solid and liquid lipids), crystallizing in a less ordered manner, thus providing more space for the entrapment of bioactive compounds. However, NLCs typically have a lower melting point than SLNs, which limits their application in products subjected to thermal treatment. The production method directly impacts the characteristics of the produced LNs, and therefore, methods that ensure the production of stable nanoparticles with potential for industrial scaling should be chosen.

**Figure 1:**
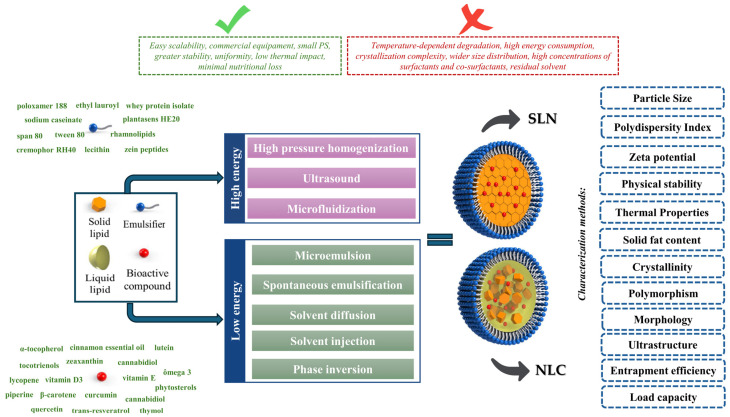
Schematic diagram of the review.

Based on this, this review provides a comprehensive and up-to-date overview, taking into account research published in the field over the past 15 years, of the chemical, physical, and structural properties of lipids used in the production LNs, as well as the formulation components, including lipid matrices, emulsifiers, and bioactive compounds (Please see Figure 1). It also offers an in-depth analysis of production methods, consolidating insights from the scientific literature, and emphasizes the most relevant characterization techniques for LNs, focusing on parameters such as particle size (PS) and distribution, zeta potential (ZP), crystallinity and polymorphism, thermal behavior, solid fat content, morphology, ultrastructure, entrapment efficiency (EE), load capacity (LC), and physical stability. Finally, the review aims to identify key factors for the successful production of stable LNs that can efficiently encapsulate and deliver bioactive compounds, thereby demonstrating significant potential for application in food systems.

## 2. Lipid Nanoparticles

### 2.1. Lipids and Crystallization Properties

Lipids are water-insoluble (hydrophobic) substances that can be derived from animal, vegetable, or microbial sources. From a chemical perspective, natural oils and fats are intricate blends of triacylglycerols (TAGs), which are synthesized through the esterification between glycerol and fatty acids (FAs). Each FA can be positioned at one of three sites on the glycerol molecule (*Sn*−1, *Sn*−2, or *Sn*−3), leading to a diverse range of possible combinations. The physical properties of lipids are determined by the characteristics of the FAs’ alkyl chains, including factors such as chain length, degree of saturation, and the configuration of double bonds (cis or trans). Saturated fatty acids (SFAs), which lack double bonds, are less reactive and have higher melting points (MPs) compared to their unsaturated counterparts of the same chain length. TAGs composed of FAs with saturated and long chains exhibit a high MP, primarily due to the linear structure of SFAs, which facilitates molecular interactions and the packing of the FAs’ chains [10].

The crystallization process involves the self-organizing of a lipid matrix, marked by a partial or complete limitation of molecular mobility due to chemical or physical interactions among TAGs molecules. Variations in crystal forms arise from differences in the molecular packing arrangements. A crystal, by definition, is composed of molecules that are systematically organized into a fixed, repeating pattern called a lattice. The inherent molecular complexity of TAGs enables them to form multiple distinct and relatively stable crystalline structures from the same molecular composition [11]. Compounds with long chains, such as FAs and their esters, can exhibit different crystalline forms, which are referred to as polymorphs. Polymorphism refers to the capacity of a substance to adopt various unit cell structures, resulting from different ways the molecules are packed together [12]. Three primary polymorphic forms are commonly observed in lipids: α, β’, and β. The α form is metastable and features hexagonal chain packing; the β’ form exhibits intermediate stability with orthorhombic packing in a perpendicular arrangement; and the β form is the most stable and exhibits triclinic parallel packing [11,13]. As the stability of these polymorphs increases, so does their melting temperature, which is attributed to variations in molecular packing density [13]. TAGs normally crystallize first in the α and β’ forms, with the β form ultimately being the most stable. This behavior is attributed to the fact that the β polymorph has a higher activation free energy for nucleation, making its formation more thermodynamically favored over time. Polymorphic transformation is a non-reversible process in which a lipid system transitions from a less stable to a more stable form, a phenomenon known as monotropic phase transformation. This process is influenced by both temperature and time. Under constant temperature conditions, the α and β’ forms of TAGs can progressively transition into the β form, either via liquid–solid or solid–solid mechanisms, depending on the transformation duration [14]. The rate of this transformation diminishes as the heterogeneity within the TAGs increases [11].

The physical properties of oils and fats are primarily defined by their solid–liquid and liquid–solid phase transitions, which correspond to melting and crystallization, respectively. These thermal phenomena are observed by monitoring changes in enthalpy and the transitions of phase in different TAG mixtures [15]. Crystallization induces volume contraction and is associated with an exothermic effect, while melting causes volume expansion, which is characterized by an endothermic effect. Thus, the thermal behavior of lipids is a key indicator of their functional properties and potential applications [16,17].

### 2.2. Solid Lipid Nanoparticles (SLNs) and Nanostructured Lipid Carriers (NLCs)

LNs were introduced as an alternative delivery system to polymeric structures, emulsions, microparticles, and liposomes. The original concept behind lipid-based colloidal suspensions involved adapting the principles of oil/water emulsions stabilized by phospholipids to a submicron scale, replacing the liquid oil phase with solid TAGs. The first documented instance of LNs development was a patent application in 1990. Since then, numerous research groups have contributed to the advancement of various methods for the preparation of stable LNs [18,19,20] (Please see Section 4).

In comparison to conventional encapsulation systems, lipid-based delivery systems offer several advantages, such as the ability to be formulated using natural ingredients on an industrial scale, significant variation in physicochemical properties, and the capability to encapsulate compounds with diverse solubility profiles [7,8]. LNs are regarded as complex, prolonged-delivery systems exhibiting spherical morphology and dimensions at the nanometer scale, though their characteristics can vary depending on the formulation and production methods. These nanoparticles demonstrate high bioavailability and low citotoxicity due to the use of physiological lipids [21,22]. Typically, LNs range in size from 50 to 1000 nm, are physiologically compatible, and present MPs higher than both room and body temperatures [9,23,24].

In comparison to conventional colloidal systems, such as microemulsions, LNs offer several distinct advantages: they are free from biotoxicity, enable controlled release and targeted delivery of substances, enhance the stability of the encapsulated compounds, and can incorporate both lipophilic and hydrophilic substances at high concentrations [25]. The significant potential of LNs lies in their remarkable versatility, enabling the design of systems with customized physical properties, including size, structure, surface charge, physical state, and preferential polymorphism. These characteristics can be precisely controlled by selecting specific lipid raw materials in combination with various production techniques [15,17].

Lipid-based nanoparticles are currently classified into two main categories: SLNs and NLCs. SLNs are produced based on the concept of replacing the aqueous core of emulsions with solid lipids. The first generation of LNs, SLNs, were produced only with solid lipid matrices and emulsifiers. Subsequently, the second generation, known as NLCs, was developed by incorporating a combination of solid lipids, liquid lipids, and emulsifiers. This advancement aimed to create a less structured crystalline lipid matrix, enhancing EE [8,24,26,27].

SLNs consist of a core of solid lipids (high MP) that possess a highly ordered crystalline structure, where the encapsulated compound is integrated into the lipid matrix. Surrounding the particle, SLNs feature an external layer composed of emulsifiers, either alone or in combination, which stabilize the structure [15,23]. The lipid phase of SLNs can be composed with an individual solid lipid matrix, such as high MP TAGs, or a combination of different lipid classes. The physical state and the structural configuration of the lipid system govern the mobility of the entrapped component [28,29]. Both lipophilic and hydrophilic bioactive compounds can be entrapped within the SLNs structure, where they are protected from degradation processes by the lipid matrix [15]. The successful development of SLNs requires a thorough understanding of the chemical interactions between the encapsulated compound and the lipids used in formulation. This includes considering the miscibility of the encapsulated compound with the lipid in its liquid state (melted), the chemical composition and physical properties of the lipid phase, and its preferential polymorphic form [30,31].

In certain instances, SLNs formed from solid lipid matrices can crystallize in a highly ordered manner, leaving limited space for the encapsulated compound. This results in stability issues and the potential expulsion of the bioactive compound. To address these challenges, NLCs were developed by combining lipid molecules with different spatial properties, aiming to produce particles with imperfect crystallization characteristics [8,15,32]. The production of NLCs involves the combination of both solid and liquid lipid fractions. Due to the imperfections in the crystalline structure of NLCs, their entrapment capacity is enhanced, thereby minimizing the release of the active compound during storage [24,33,34].

**Figure 2:**
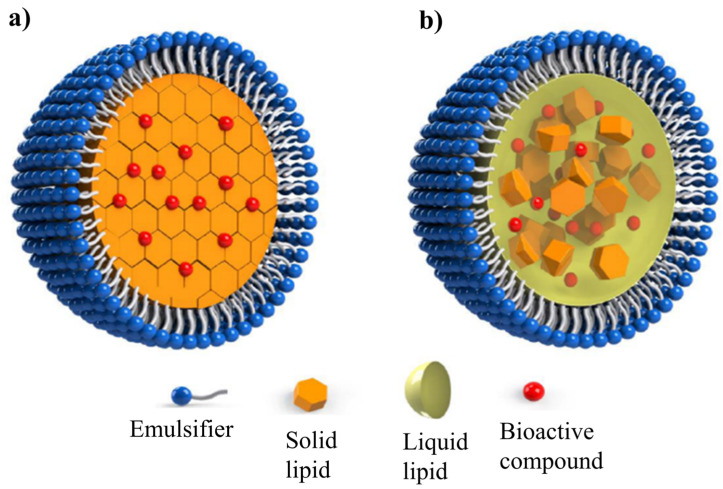
Schematic representation and differences between (**a**) SLNs and (**b**) NLCs.

The lipid matrices employed in the formulation of NLCs should exhibit a low polymorphic transition rate and a reduced degree of crystallinity, properties typically associated with the presence of liquid lipid domains within the particle core [29]. Generally, crystal imperfections can be enhanced by using mixtures of TAGs or acylglycerols (mono- and diacylglycerols) composed of FAs that differ in chain length and degree of saturation. Structural incompatibility among the lipid components can be achieved by selecting lipid sources with distinct physicochemical characteristics, such as high-MP TAGs and liquid vegetable oils, thus promoting the desired imperfections for improved NLCs performance [17,35]. Figure 2 provides a schematic representation of the structural characteristics of LNs types: SLNs and NLCs.

## 3. Lipid Nanoparticle Formulation

The key components for the preparation of SLNs and NLCs include lipids, emulsifiers, and water. These lipid nanoparticles typically contain lipid concentrations ranging from 0.1% to 30% (*w*/*w*) and emulsifier concentrations between 0.5% and 5% (*w*/*w*). The molten lipid matrices are generally dispersed in an aqueous medium and stabilized by emulsifiers. In both systems, the encapsulated compound typically constitutes approximately 5% (*w*/*w*) of the total formulation. The careful selection of lipids, emulsifiers, and their respective concentrations significantly impacts various physicochemical properties, including PS, long-term stability during storage, and the loading and release behavior of bioactive compounds [8]. Thus, the selection of materials for the preparation of LNs should take into account, among other factors: (i) the use of ingredients recognized as GRAS (Generally Recognized as Safe), (ii) the intended application of the LNs, and (iii) their availability and economic cost. Table 1 presents some of the key ingredients used in the preparation of LNs in recent studies.

### 3.1. Lipids

The lipid matrix plays a pivotal role in determining the structure and properties of LNs, as it governs the molecular configurations directly linked to the functionality of these nanostructures. A thorough understanding of the chemical composition, crystal structure, and thermal behavior during melting and crystallization is essential for the development of LNs. Factors such as the crystallization rate of the lipid phase and its structural properties significantly influence the overall characteristics of LNs, including their shape, size, the entrapment and release of bioactive compounds, and their behavior in the gastrointestinal tract (GIT), ultimately affecting their fate.

Regarding particle production, the lipid matrix plays a crucial role in influencing the system’s viscosity, which directly impacts the characteristics of the resulting particles. The high MP of lipids increases the viscosity of the dispersed phase, thereby reducing the effective force resulting from the homogenization and sonication processes, leading to an increase in PS [36,37]. Furthermore, the production conditions of lipid nanoparticles, such as homogenization temperature and specific cooling rates, are determined by the lipid matrix [4].

The physical state and FAs composition of lipid matrices used in LNs formulation significantly influence lipid digestion rates and the bioaccessibility and bioavailability of entrapped bioactive compounds. Medium-chain FAs typically exhibit a faster formation of free fatty acids (FFAs) in greater amounts compared to long-chain FAs [38,39]. This behavior is attributed to the rapid migration of medium-chain FFAs to the aqueous phase, whereas long-chain FFAs tend to accumulate at the oil-water interface, resulting in slower digestibility [40]. The lipid matrix composition also affects the solubilization capacity of mixed micelles. Longer-chain FAs with fewer unsaturations increase this capacity, allowing better incorporation of bioactive compounds [38]. The degree of FA saturation also influences bioactive compound solubilization. Studies show that the release of long-chain monounsaturated FAs, like oleic acid, enhances micelle solubilization and bioaccessibility compared to polyunsaturated FAs [40,41]. The rate and extent of lipid digestion were greater in liquid particles compared to solid particles. The lower MP of the lipid matrix facilitates increased mobility and fluidity of TAG molecules at the interface, thereby enhancing lipase binding to its active site [42,43].

The selection of appropriate lipids for the development of SLNs and NLSs should take into account the following factors: (i) the solubility of the encapsulated active ingredient in the lipid phase, EE, and scalability; (ii) the stability of the lipid phase against chemical and enzymatic processes; and (iii) the use of biodegradable and non-toxic lipid components [29,44]. In addition to being biocompatible, biodegradable, and well-tolerated by the body’s physiological processes, lipids used in the synthesis of SLNs must maintain their solid state at both room and body temperatures [23]. In the case of NLCs, lipids with high and low MPs must exhibit distinct spatial configurations. Specifically, the lipids with a lower MP should not incorporate into lipids with a higher MP, and the crystals formed during cooling should remain insoluble in the liquid lipid (lower MP). Furthermore, a critical condition for ensuring the stability of NLCs is that high- and low-MP lipids must be completely miscible at the concentrations used [45].

In the case of NLCs, in addition to the type of lipid matrix, the ratio of solid to liquid lipids plays a crucial role in determining the physicochemical properties and structural characteristics of the resulting particles. These factors also influence the rate and extent of lipid digestion. For example, Ludtke et al. (2022) observed that increasing the proportion of liquid lipid in NLCs formulations led to larger PS, enhanced physical instability, and reduced peak temperature and melting enthalpy [9]. Azevedo et al. (2023) demonstrated that an increase in liquid lipid in the formulation content resulted in NLCs with a higher PS, and that the solid-to-liquid ratio in the lipid phase also influenced the rate and extent of lipid digestion and consequently the vitamin D3 bioaccessibility [46]. Barri et al. (2023), on the other side, reported that the increase in liquid lipid content in the NLC formulation resulted in a reduction of PS and polydispersity index (PDI) [47]. The authors attributed the smaller PS to the reduced viscosity and enhanced fluidity of the system, as well as to the increased presence of α and β’ crystals in the lipid matrix. Vardanega et al. (2024) observed that increasing the lipid content in unloaded NLCs did not affect PS and PDI. However, in cannabidiol (CBD)-loaded NLCs, an increase in the liquid lipid content resulted in a reduction of PS [48]. The authors proposed that the inclusion of a higher proportion of liquid lipid in the NLCs formulation enhanced the solubilization of CBD within the structure, primarily due to intermolecular interactions between CBD and the nonpolar components of the NLCs, which contributed to the formation of a more compact structure. Song et al. (2025) reported that the use of beeswax in the formulation of NLCs resulted in larger particles and lower EE compared to the use of glyceryl dibehenate [49]. This was attributed to the longer carbon chain and higher MP of beeswax.

Table 1 presents some examples of lipid matrices used to produce SLNs and NLCs. Most lipids used in the production of LNs are synthetic, incurring a high cost, and exhibit variable chemical compositions that vary according to the manufacturer. The solid lipids used in the preparation of SLNs and NLCs primarily consist of TAGs, FAs, monoacylglycerols, diacylglycerols, and waxes. Due to their compatibility with the lipid composition of animal tissues, myristic, palmitic, and stearic acids have become favored lipid matrices for the preparation of LNs [50]. Stearic acid is an endogenous long-chain fatty acid and a key component in both natural and modified oils and fats. With a MP of approximately 70 °C, it is regarded as physiologically neutral. Due to these properties, stearic acid, along with tristearin and lipid mixtures enriched in these compounds, is widely employed as a raw material in the formulation of LNs [51,52]. Natural waxes, such as carnauba, candelilla, sunflower, and rice bran waxes, are commercially available and approved as Generally Recognized as Safe (GRAS) for food use. These materials are known for their high stability against oxidation processes and can be directly employed in the formulation of these structures [26]. Additionally, the literature identifies several potential solid lipid materials for the composition of LNs, including glyceryl monolaurate, glyceryl behenate, as well as natural or acetylated monoacylglycerols and diacylglycerols [15,53]. Medium-chain TAGs (MCTs) and oleic acid are among the most commonly used components in the development of NLCs (Table 1). Oleic acid, a primary component of most edible oils and fats, is widely acknowledged for its well-established health benefits. It also demonstrates greater resistance to oxidation when compared to polyunsaturated FAs, such as linoleic and linolenic acids [10].

Therefore, the combination of the FAs’ characteristics found in common lipid sources, such as vegetable oils and fats, is highly advantageous for the development of LNs. These raw materials are commercially available on a large scale, widely consumed in human diets, and cost-effective. Moreover, the constant consumer demand for natural and safe food ingredients makes edible oils and fats well-suited for clean-label products [7]. In this context, edible vegetable oils, including soybean, sunflower, corn, and canola oils, are commonly utilized in the production of NLCs (Table 1). The addition of these natural oils modifies the crystallinity of the lipid matrix, typically enhancing the solubilization of the encapsulated components.

An interesting option for solid lipid sources to produce LNs is fully hydrogenated oils (FHOs). These materials are obtained through the complete catalytic hydrogenation of liquid oils, where all double bonds of FAs’ chains are saturated by the addition of the H^+^ ion to the double bonds. Due to their low cost and compliance with technological requirements for food applications, FHOs are commonly used as ingredients in industrial fats, making them a promising choice for nanostructured systems. Recently studied FHOs include those derived from soybean, palm, microalgae, and crambe oils. These oils are characterized by SFAs with chain lengths ranging from 12 to 22 carbon atoms, which impart distinct physical properties, thereby enabling their customization for the development of LNs with specific characteristics. Additionally, they possess MPs ranging from 40 to 72 °C, which makes them suitable for use as solid lipids in LNs [17,23].

**Table 1:** Lipid matrices, emulsifiers, and bioactive compounds used to produce LNs.

Lipid Nanostructure	Bioactive Compound	Lipid Matrices	Emulsifiers	Main Outcomes	Reference
NLCs	β-carotene and α-tocopherol	Murumuru butter	Span 80 Cremophor RH40	- The β-carotene stability was notably improved by the co-encapsulation of α-tocopherol within mumumuru-based NLCs. - The bioaccessibility of β-carotene after NLCs passage in the in vitro dynamic digestion system was approximately 42%. - The surfactant employed, in combination with the extremely small PS, rendered the produced NLCs toxic to the tested cell cultures.	[54]
NLCs	Phytosterols	High oleic sunflower oil Fully hydrogenated canola and crambe oils	Tween 80	- The entrapment of phytosterols was enhanced by the use of fully hydrogenated canola and crambe oils separately in the NLCs formulation. - The increase in the phytosterol amount within the NLCs formulation led to a higher PS. - The incorporation of phytosterols contributed to an increase in the crystallinity of the developed systems.	[55]
NLCs and SLNs	β-carotene	MCT Glyceryl stearate Hydrogenated palm oil	Tween 80	- The concentration of glyceryl stearate does not affect lipid digestibility. - Hydrogenated palm oil delays the lipolysis kinetics of SLNs. - The composition of the micelle fraction determined the concentration of β-carotene. - Monounsaturated FAs enhanced the solubilization capacity of micelles.	[40]
NLCs	Cinnamon essential oil	Corn, sesame, sweet almond, and black seed oil Cocoa butter	Tween 80	- NLCs formulated with oils with higher concentrations of SFAs (corn and sesame oils) exhibited a larger average PS. - The NLCs formulated with almond oil demonstrated the most favorable physicochemical properties and the highest encapsulation stability.	[56]
NLCs	Quercetin and piperine	Glyceryl behenate Squalene	Tween 80 and Span 80	- The NLCs exhibited a negative surface charge and an average PS of less than 200 nm. - The EE for both quercetin and piperine exceeded 90%. - The safety of the NLCs was demonstrated by the absence of hemolysis in blood samples.	[57]
NLCs and SLNs	Lutein	Cocoa butter Corn oil	Zein peptides Tween 80	- SLN samples exhibited precipitation and during the storage period. - SLN system exhibited the highest FFAs release rate and lutein bioavailability.	[58]
NLCs	Lycopene	Cocoa butter Grape seed oil	Span^®^ 80 Plantasens^®^ HE20	- An increase in the emulsifier amount (from 2.5% to 7.0%) resulted in NLCs with smaller PS and higher ZP. - The NLCs formulation effectively protected lycopene from instability during storage, particularly at 4 °C for a duration of 3 months.	[59]
NLCs	Phytosterols	Soybean oil Fully hydrogenated palm and crambe oils (simple and interestefied)	Enzymatic modified soybean lecithin	- NLCs formulated with interesterified lipid blends exhibited greater stability compared to those developed with single lipid blends. - Interesterification led to a reduction in the MP and recrystallization index of the NLCs. - The highest EE and LC were observed in NLCs formulated with interesterified lipid blends.	[60]
NLCs	Thymol	Fully hydrogenated palm oil Thymol oil	Tween 80	- The increase in thymol content in the NLCs formulations did not affect their physical properties. - The NLCs provided a slower release of thymol in active calcium alginate films, with the release rate being slower in the structures containing a higher thymol content. - The NLC-based films demonstrated sustained antimicrobial activity in the model food system (ground beef) over extended periods, attributable to their slow-release profiles.	[61]
SLNs and NLCs	Zeaxanthin	Glycerol monostearate and Glycerol distearate MCT	Tween 80 and lecithin	- SLNs exhibited larger PS compared to NLCs. - NLCs exhibited higher EE and LC than SLNs. - The entrapment of zeaxanthin increased the PS of both SLNs and NLCs. - The use of glycerol distearate decreased the viscosity of the dispersed phase of LNs, resulting in a more uniform PS distribution compared to glycerol monostearate.	[37]
NLCs	Vitamin D3	Glycerol monostearate MCT	Rhamnolipids	- An increase in the liquid lipid content resulted in NLCs with larger PS. - The ratio between solid and liquid lipid influenced both the rate and extent of lipid digestion.	[46]
NLCs	Vitamin D3	Anhydrous milk fat Vitamin E acetate	Sodium caseinate	- The PS and PDI increased as the caseinate concentration reached 3%. - The increase in liquid lipid content in the NLCs formulation resulted in a reduction of PS. - The incorporation of vitamin D3 into the NLCs structure enhanced its anticancer effect.	[47]
SLNs	Vitamin E tocotrienols	FAs esters	Poloxamer 188	- SLNs facilitated the increased bioavailability and selective tissue accumulation of tocotrienol.	[62]
NLCs and SLNs	Curcumin	Beeswax MCT	Lecithin Tween 80	- Both NLCs and SLNs demonstrated good PS stability within the beverage throughout the storage period. - The beverage containing NLCs exhibited slightly greater color stability. - The SLNs-based beverage exhibited higher curcumin bioaccessibility compared to the NLCs-based beverage, although it showed reduced curcumin stability.	[63]
NLCs	β-carotene	Fully hydrogenated soybean oil High oleic sunflower oil	Tween 80 Enzymatic modified soybean lecithin Whey protein isolate (WPI)	- The β-carotene entrapment into NLCs structure altered the polymorphic habit of NLCs and increased the energy required for melting. - WPI-based NLCs showed larger PS, lower physical stability, and reduced EE and LC.	[64]
NLCs	Lutein	Glyceryl distearate MCT	Ethyl lauroyl arginate Rhamnolipid Tea saponin	- Emulsifiers played a regulatory role in the crystallization behavior of NLCs. - Rhamnolipid-stabilized NLCs demonstrated higher EE for lutein, the slowest release of FFAs, and exhibited high bioaccessibility.	[65]
NLCs	β-carotene	Fully hydrogenated soybean oil High oleic sunflower oil	Tween 80 Enzymatic modified soybean lecithin	- NLCs stabilized with Tween 80 exhibited superior physical stability during passage through the in vitro dynamic GIT system, leading to an increase in β-carotene bioaccessibility. - Both NLCs systems did not affect cell viability up to a β-carotene concentration of 25 μg. mL^−1^.	[21]
SLNs	Omega-3 Vitamin D3	Beeswax	Egg yolk lecithin Tween 80	- The addition of vitamin D3 to the formulations decreased the NLC PS. - The EE of VD3 increased when ω-3 was included in the formulations. - SLNs effectively protected both bioactives compounds against oxidation and high pH levels.	[66]
SLNs	α-tocopherol	Fully hydrogenated palm, soybean and crambe oils	Enzymatic modified soybean lecithin	- The entrapment of α-tocopherol modified the thermal behavior of the particles, resulting in increased crystallinity without affecting the polymorphic structure. - α-Tocopherol-loaded SLNs dispersions demonstrated stability, with no significant loss of α-tocopherol.	[30]
NLCs	Phytosterols	Walnut oil Stearic acid	Soybean lecithin	- The bioaccessibility of phytosterols significantly increased (four times) when entrapped into NLCs. - The incorporation of phytosterols also effectively reduced lipid oxidation of the NLCs.	[67]
NLCs	Cannabidiol	Hemp seed oil Fully hydrogenated soybean oil	Tween 80 Enzymatic modified soybean lecithin	- The use of individual emulsifiers to produce NLCs resulted in smaller PS; - Increasing the proportion of liquid lipid in unloaded NLCs did not significantly affect the PS or PDI. - An increase in the liquid lipid content in CBD-loaded NLCs led to a reduction in PS. - The CBD retention rate remained at 100% over a period of 30 days.	[48]
SLNs	Vitamin D3	Beeswax	Lecithin	- SLNs demonstrated high EE for vitamin D3, achieving a high concentration. - The enrichment of drinks with SLN-Vitamin D3 did not exhibit any negative effects on cell viability. - The supplementation of thickened beverages with SLNs proved to be a viable strategy, without significant impact on the rheological properties of the beverages.	[68]
NLCs	Emodin	Beeswax Glycerol di-stearate MCT	Tween 80	- The incorporation of beeswax in the formulation of NLCs resulted in larger particles and lower EE compared to the use of glyceryl dibehenate. - The EE of emodin was not significantly influenced by the solid and liquid lipid ratio in the NLCs.	[49]

Interesterified fats represent a promising lipid material for NLCs formulation. This lipid modification facilitates the redistribution of FAs within TAGs, resulting in changes to physical properties, crystallization patterns, melting behavior, and polymorphism. As a result, the incorporation of interesterified lipids in NLCs development offers a potential strategy for creating lipophilic nanocarriers with reduced crystalline structure, improved stability, and suitable polymorphism, which is essential for the effective entrapment of bioactive compounds like phytosterols [60,69].

In addition to the proper selection of lipid matrices for the formulation of LNs, the characterization of the physical properties and crystallization behavior of these matrices at a macroscopic scale is essential for optimizing the formation, stability, and functionality of LNs [17,70]. Therefore, any study on the development of LNs should incorporate this step to ensure the formulation of LNs with appropriate crystallinity, which is critical for the effective incorporation of bioactive compounds.

### 3.2. Emulsifiers

The selection of emulsifiers and their respective concentrations significantly influences the properties of LNs. Emulsifiers are surface-active molecules that adsorb at the oil–water interface, reducing interfacial tension between the two phases due to their amphiphilic nature [24]. Structurally, emulsifiers are composed of a hydrophilic head group, which has a strong affinity for water, and a hydrophobic tail group, which interacts predominantly with the oil phase [71]. Several factors should be considered when selecting the most appropriate emulsifier for the lipid matrix and the bioactive compound to be encapsulated in LNs. These factors include the type and composition of head group and tail group, polarity and charge, molecular geometry, solubility, surface activity, and adsorption kinetics [72]. The affinity of an emulsifier for the oily or aqueous phase is often characterized by its hydrophilic–lipophilic balance (HLB), an index ranging from 0 to 20 that estimates the emulsifier’s hydrophilicity. The HLB value can be calculated based on the type and quantity of hydrophilic and lipophilic groups, and the HLB can be adjusted by combining two or more emulsifiers with different HLB values. Another important classification of emulsifiers is based on the charge of the head group, which can be anionic, cationic, zwitterionic, or non-ionic [73].

Table 1 presents some examples of emulsifiers used to produce LNs. The most commonly used emulsifiers for the preparation LNs include polyoxyethylene sorbitan monooleate (commonly known as Polysorbate^®^ 80 or Tween^®^ 80), lecithins, sorbitan monostearate (Span), Poloxamer 188 (a polyoxyethylene and polyoxypropylene copolymer), mono- and diacylglycerols, sucrose esters, polyglycerol esters, and lactate esters [15].

The number of emulsifiers approved for food applications remains limited, making the development of nanostructures using natural emulsifiers a significant challenge in the production of LNs [20,44]. Natural emulsifiers explored for LNs development include protein-based compounds, such as whey protein, casein, and soy protein, as well as polysaccharide-based emulsifiers like gum arabic, xanthan gum, pectin, and alginate. Among the natural emulsifiers derived from smaller molecules are lecithins, which can be obtained from sources such as soybeans, sunflower, milk, eggs, and canola. Furthermore, lecithins can be modified chemically or enzymatically to cleave one of the FAs’ tails, producing lysolecithins, which possess a more hydrophilic character [23,74].

In conventional emulsions, the emulsifier adsorbs at the oil–water interface, providing repulsive forces that are sufficient to prevent flocculation or coalescence. This primarily influences the PS achieved during homogenization and the stability of the dispersion. In the production of LNs, emulsifiers assume an additional crucial role by acting as crystallization modulators of the lipid phase. Due to the small size of LNs, there is a greater interaction between the emulsifier’s functional groups and the lipid phase, which in turn modulates the crystallization kinetics. Moreover, emulsifiers can influence the natural polymorphic form of the lipids, helping to minimize issues related to recrystallization and destabilization of LNs during storage [10].

The production of LNs involves various methods that require specific combinations and concentrations of emulsifiers, to stabilize the particles. These emulsifiers are incorporated into either the lipid or aqueous phase depending on their HLB value. The optimal concentration of emulsifier in LNs formulations is contingent on the lipid matrix used. The emulsifier must be present in sufficient quantity to form a protective layer around the lipid phase once the particles are generated. Generally, the success of the emulsification process in producing LNs depends on the rate at which the emulsifier coats the particle surface and its interaction with the lipid components. Ideally, the production of LNs should result in a gradual increase in the number of droplets, with immediate emulsifier coverage to effectively stabilize them [19]. Therefore, when selecting an emulsifier for LNs preparation, it is crucial to consider factors such as the emulsifier’s physicochemical and structural properties, availability, toxicity, compatibility with the lipid phase, and with the bioactive compound to be incorporated, and its stability under GIT conditions.

The type of emulsifier can significantly impact the bioavailability of bioactive compounds by influencing lipase adsorption on the particle surface. In the presence of bile salts, lipase adsorbs to the surface, initiating the lipolysis reaction. However, certain emulsifiers cannot be fully displaced by bile salts, which inhibits lipase adsorption and consequently reduces lipid digestion [75]. Additionally, some emulsifiers exhibit antioxidant properties, enhancing bioavailability by preserving the stability of bioactive compounds [76]. Furthermore, emulsifiers can either facilitate or hinder the formation of mixed micelles, which are essential for solubilizing bioactive compounds after their release from the lipid matrix, thereby influencing their bioavailability [77].

To enhance the efficiency of certain emulsifiers in the production of LNs, co-emulsifiers can be utilized. These molecules, which contain polar components in their structure, such as alcohols and short-chain organic acids, modify the physicochemical properties of the emulsifiers. Co-emulsifiers function by increasing entropy at the oil–water interface, thereby destabilizing crystalline structures and increasing the viscosity of the dispersions [71].

Recent studies have explored the effects of individual emulsifiers on the particle characteristics, physicochemical stability, and functionality of LNs (Table 1). For example, Shu et al. (2023) investigated the use of ethyl lauroyl arginate, rhamnolipid, and tea saponin to stabilize lutein-loaded NLCs [65]. The authors found that rhamnolipid-stabilized NLCs exhibited the highest EE, the slowest release of FFAs, and optimal sustained release for lutein, along with relatively high bioaccessibility. Ludtke et al. (2023) compared the use of Tween 80, enzymatically modified soybean lecithin, and WPI to produce β-carotene-loaded NLCs [64]. Their findings indicated that WPI resulted in NLCs with larger PS, lower physical stability, and reduced EE LCs compared to the other emulsifiers. Recently, Ludtke et al. (2024) assessed the performance of β-carotene-loaded NLCs produced with Tween 80 and enzymatically modified soybean lecithin under dynamic in vitro digestion conditions [21]. The results showed that NLCs stabilized with Tween 80 demonstrated superior physical stability during in vitro dynamic GIT system compared to those stabilized with enzymatically modified soybean lecithin. This enhanced stability resulted in better protection and delivery of β-carotene, as evidenced by a tenfold increase in β-carotene bioaccessibility. Vardanega et al. (2024) investigated the influence of soybean lecithin (SL), Tween 80 (T80), and a 50:50 mixture of SL and T80 on NLCs production [48]. The NLCs stabilized with T80 (T80:SL 100:0) exhibited the smallest PS, followed by those stabilized with SL. In contrast, the NLCs produced with the combined emulsifiers (T80:SL 50:50) had the largest PS. The authors attributed the increase in PS to structural differences between T80 (a non-ionic emulsifier) and SL (a zwitterionic emulsifier), which may have resulted in less efficient coverage of the droplets during the NLCs production process, leading to larger particles compared to those stabilized by each emulsifier individually.

Several studies have also examined the effects of emulsifier concentration on the characteristics of LNs. Sirikhet et al. (2021) produced lycopene-loaded NLCs using a 50:50 mixture of Span 80 and Plantasens (1:1) and found that increasing the emulsifier content from 2.5% to 7.0% resulted in NLCs with smaller PS and higher ZP [59]. Barri et al. (2023) investigated the impact of sodium caseinate concentration (1% to 3%) on the size, PDI, and EE of vitamin D3-loaded NLCs [47]. They observed that increasing the caseinate concentration from 1% to 3% led to an increase in PS and PDI across all liquid/total lipid ratios, while EE was higher when the sodium caseinate concentration was below 2% (*w*/*v*). In another study, Vardanega et al. (2024) explored the use of ionic liquid (IL) as an emulsifier at concentrations of 1% and 2% for NLC production [22]. They found that 2% IL resulted in NLCs with smaller PS, whereas the 1% IL concentration produced NLCs with excellent stability and high CBD retention during storage. Furthermore, the 1% IL concentration provided greater stability to CBD during in vitro digestion. Although many of these studies have indicated that a higher emulsifier concentration results in LNs with improved characteristics, the lowest possible emulsifier concentration that ensures stable LNs should be chosen, due to regulatory and safety concerns, as well as the need to produce clean label products.

### 3.3. Lipophilic Bioactive Compounds

Bioactive compounds, naturally found in plants and certain foods, offer distinct health benefits and contribute to disease prevention. Their antioxidant, anti-inflammatory, and disease-modulating properties underscore their significance in promoting a balanced diet [7]. However, many of these compounds exhibit lipophilic characteristics, which often result in poor stability, solubility, and bioavailability when present in their free form [24].

LNs are increasingly recognized as optimal systems for encapsulating bioactive compounds due to their toxicological safety, scalability, and technological performance [15,24,53]. The utilization of LNs for the delivery of functional or bioactive compounds has emerged as a key focus in food technology, particularly for controlled release, protection during industrial processing, and efficient delivery. Owing to their nanometric size, these systems can enhance the solubility and bioavailability of encapsulated compounds, while also preventing undesirable chemical reactions and facilitating the controlled release of components, particularly those with low solubility in aqueous environments [60,78].

Bioactive compounds exhibit considerable variation in their molecular properties, including molecular weight, structure, functional groups, polarity, and charge. These differences lead to distinct physicochemical and physiological properties, such as solubility, physical state, rheological behavior, chemical stability, and functionality. Consequently, each delivery system is defined by its own set of unique properties. Among the primary lipophilic bioactive compounds studied for incorporation into LNs are carotenoids, antioxidants (such as tocopherols and polyphenols), fat-soluble vitamins (A, D, E, and K), phytosterols, omega-3 FAs, essential oils, and antimicrobial agents (Table 1). Recently, NLCs have also been successfully utilized for the entrapment and delivery of CBD [22,48].

Co-encapsulation of two or more bioactive compounds is a promising strategy to enhance their functionality and bioactivity, often leading to synergistic effects that may be more beneficial than the use of a single bioactive compound [66]. In this context, Gomes et al. (2019) demonstrated that the stability of β-carotene was significantly enhanced by the presence of co-encapsulated α-tocopherol in mumumuru-based NLCs [54]. After 120 days, approximately 73% of the initial β-carotene mass was preserved in the NLCs containing α-tocopherol. In contrast, the NLCs without α-tocopherol showed much lower protection, with only 34% of the initial β-carotene retained after the same storage period. Similarly, Shakeri et al. (2024) found that co-encapsulating omega-3 FAs and vitamin D3 in beeswax SLNs enhanced the EE of the lipophilic vitamin [66].

## 4. Production Methods

**Figure 3:**
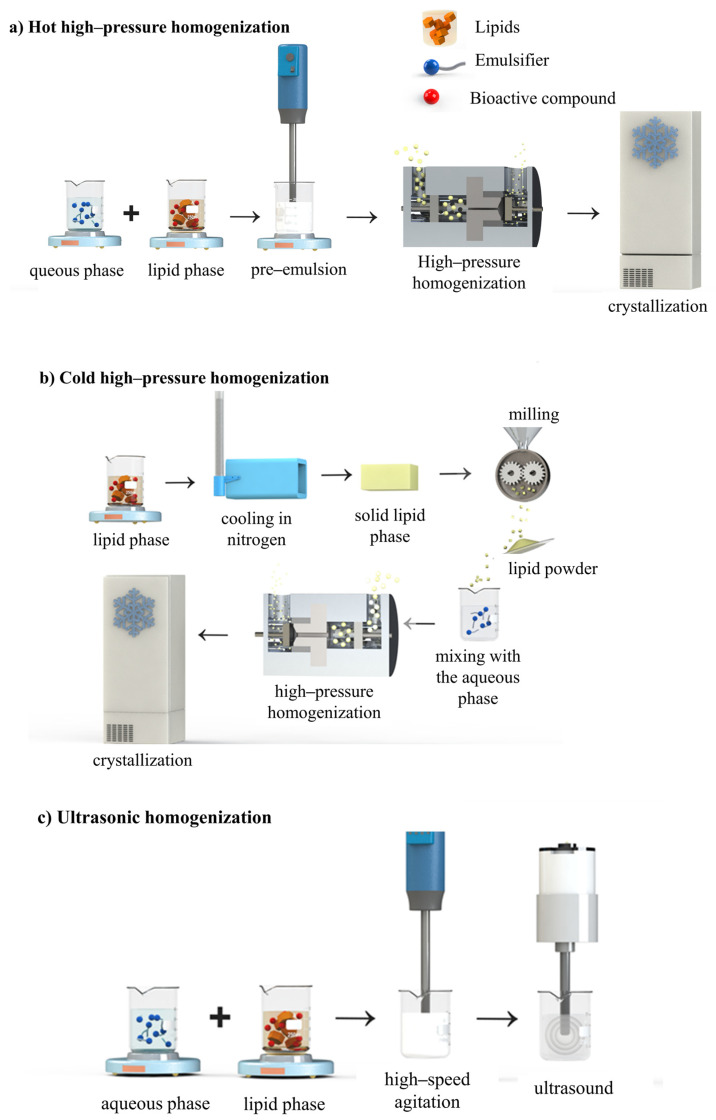
High-energy methods to produce lipid nanoparticles. (**a**) Hot high-pressure homogenization, (**b**) cold high-pressure homogenization, and (**c**) ultrasonic homogenization.

**Figure 4:**
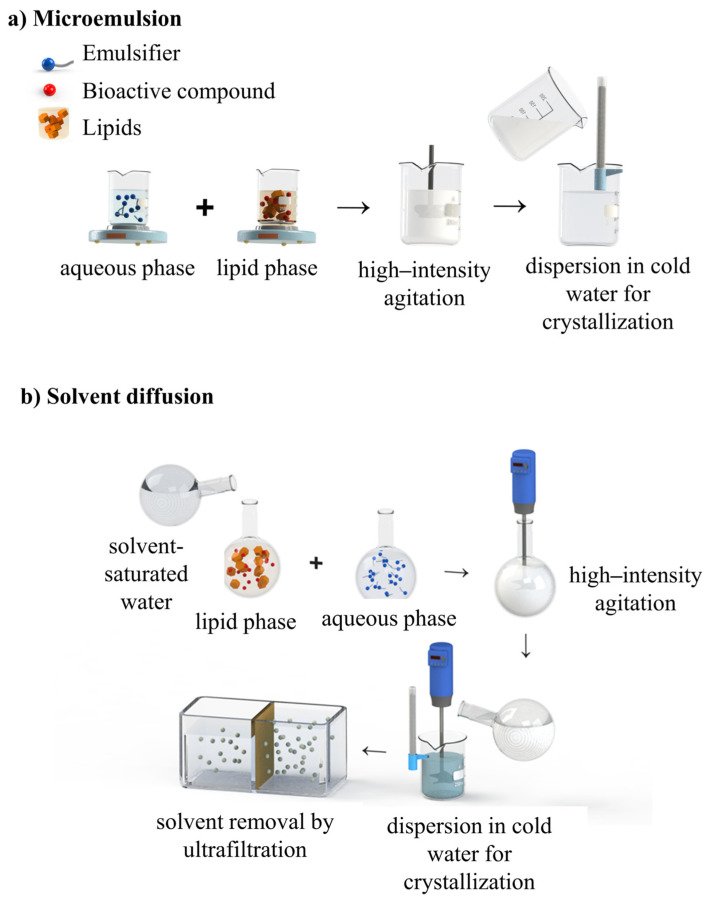
Low-energy methods to produce lipid nanoparticles. (**a**) Microemulsion and (**b**) solvent diffusion.

LNs are produced by two main groups of methods, classified according to the amount of energy required: (i) using high energy, which is provided by equipment as high-pressure homogenizers (hot or cold), high-speed homogenization, and ultrasound (Figure 3); (ii) using low energy, which includes the use of mixing processes under low agitation with a magnetic stirrer, microemulsions, phase inversion temperature, and sol-vent-based methods (diffusion and solvent injection) (Figure 3 and Figure 4). These methods are based on the following technological approaches: (i) creation of an oil-in-water nanoemulsion; (ii) solidification of the dispersed lipid phase. These methods vary substantially in scale but have a relatively low cost due to the configuration of the equipment required [15,65,79]. Table 2 presents the methods for preparing LNs, and examples of studies that used them for the entrapment of bioactives.

**Table 2:** Application of LNs in food using different manufacturing techniques.

	Method	Lipid Nanostructure	Process	Lipid Phase	Emulsifier/Surfactant	Bioactive	Reference
**High energy**	High-pressure homogenization	SLNs	700 bar and 2 cycles.	Fully hydrogenated palm, soybean, crambe oils.	Lecithin	α-tocopherol	[30]
		NLCs	700 bar and 2 cycles.	Palm, soybean, microalgae, and crambe harfat, soybean oil, interesterified lipid.	Lecithin	-	[60]
		NLCs	500 bars for 6 cycles	Tristearin, Phosal 53 MCT (lecithin 53% and MCT).	Tween 80	Ondansetron hydrochloride	[79]
	High-pressure homogenization + Microfluidization	NLCs	Microfluidizer under 50 MPa for three times.	MCT, glyceryl palmitostearate	Rhamnolipid	Emulsifier	[65]
	Microfluidization	SLNs	Pre-emulsion and homogenization in a microfluidizer (40–45 °C) for five times at 5000–28,500 psi.	Tripalmitin, cetyl palmitate, pluronic F68	Lecithin, tween 80	Trypsin, testosterone	[80]
		Complex nanoparticles	100 MPa for 2 cycles.	MCT	Tea saponin	β-carotene, curcumin	[81]
	Ultrasonic homogenization	SLNs and NLCs	Ultrasonic probe with 25% and 50 cycles (4 s on and 1 s off).	MCT	Lecithin, tween 80	Zeaxanthin	[37]
		NLCs	Ultrasonic for 5 min, amplitude intensity of 60%.	Dynasan 116, capryol 90, lauroglycol 90, miglyol 810, tributyrin	Twen 80, lecithin	Trans-resveratrol	[82]
		SLNs	100 W of ultrasonic for 3 or 5 min, amplitude 50–100% in a continuous operation or pulse modulation.	Glyceryl monostearate	Lipoid S75 (fat-free soybean phospholipids with 70% phosphatidylcholine), tween 60.	-	[83]
		SLNs	Sonication (90 W) for 2–10 min at 75 °C.	Stearic acid, tripalmitin	Span 80, tween 80	Curcumin	[84]
**Low energy**	Microemulsion	SLNs	Dispersion of hot O/W microemulsion in cold water (2–4 °C) at a 1:50 ratio (microemulsion: water).	Glyceryl monostearate, glyceryl trimyristate, glyceryl behenate, glyceryl palmitostearate, hydrogenated coco-glycerides, cetyl palmitate and stearic acid.	Tween 20	Tetracycline	[85]
		SLNs	Cold dilution of microemulsion.	Trimyristin, tristearin, myristic acid, glyceryl dibehenate, and glyceryl monostearate	Epikuron 200, polysorbate (20, 40, 80), cremophor.	-	[86]
		SLNs	Hot microemulsion dispersed in 5 parts (*v*/*v*) of cold water (2–4 °C).	Propylene glycol monopalmitate, glyceryl monostearate	Tween 80	-	[87]
	Spontaneous emulsification	SLNs	Emulsifier at 85 °C for 30 min at 500 rpm. Added to aqueous phase over 5 min at 750 or 1050 rpm.	Beeswax, propolis wax	Tween 80	-	[88]
		LNC	Under stirring. Removal of organic solvents by evaporation and concentration of colloidal dispersions.	MCT	Hydrogenated soybean lecithin, egg lecithin, tween 80	*Jatropha isabellei*	[89]
	Solvent diffusion	rLNP	Lipids dissolved in ethanol at 10 mg/mL, flow rate of 0.1 mL/s, using a syringe. Homogenization of the mixture at 500 rpm.	MCT	-	Curcumin	[90]
		NLCs	Dissolved in acetone and added to the aqueous phase (1:2 *v*/*v* ratio).	Stearic acid, Capryol PGMC, cholesterol, triolein	Brij 35, Brij 72	Simvastatin, a 3-hydroxy-3-methylglutaryl coenzyme A	[91]
	Solvent injection	NLCs	All weighted directly, and the final volume adjusted to 1 mL. 1000 rpm + 65 °C + ultrasonic probe (60 W for 90 s)	Precirol^®^ ATO 5, copaiba oil	Tween 80	-	[92]
		NLCs	Hypodermic needle syringe. Mixture stirred at 1500 rpm for 1 h (evaporation of ethanol), and cooled for 30 min.	Stearic acid to oleic acid	Sodium lauryl sulfate	Fexofenadine HCL	[93]
	Phase inversion	NLCs	The hot dispersion (murumuru butter and surfactants) was submitted to two heating/cooling cycles.	Murumuru butter	Span 80, Cremophor RH	α-tocopherol, β-carotene	[54]
		SLNs	Oil, surfactant, and water were heated at 50 °C.	Octadecane	Brij 30, C_12_E_4_	-	[94]

SLNs: solid lipid nanoparticle; NLCs: nanostructured lipid carrier; rLNP: reconstituted lipid nanoparticle; LNC: lipid nanocarrier.

### 4.1. High-Energy Methods

#### 4.1.1. High-Pressure Homogenization

High-pressure homogenization (HPH) is one of most promising methods for preparing LNs, with commercially available homogenizers in different capacities. HPH offers easy scalability and enables operation under aseptic conditions, making it highly versatile and advantageous. This technique operates using variable pressure between 100 and 2000 bar, where shear forces and cavitation break the particles on a nanometric scale [95,96,97]. The same energy intensity must be applied to LNs to obtain a homogeneous droplet size distribution. Otherwise, the lipid materials distributed throughout the sample will experience varying dispersion forces, resulting in particles with very heterogeneous dimensions. HPH is therefore characterized by the application ensuring that the entire sample experiences the same shear stress due to the small dimensions of the homogenizer outlet orifice (smaller than 30 µm) [96,98]. SLNs and NLCs can be produced by two HPH methods: hot homogenization and cold homogenization. In both cases, a preparatory step involves the incorporation of the compound to be encapsulated in the lipid matrix, by dissolving or dispersing it in the lipid phase [99].

In hot HPH, the lipid matrix is completely melted at a temperature above its MP. Before being subjected to high pressure, a mixture, known as a pre-emulsion, is obtained, and then this phase is emulsified in an aqueous phase using mechanical agitation [100]. The final quality of the LNs depends on the pre-emulsion, and particles measuring a few micrometers in size should be formed [99]. Then, the pre-emulsion is subjected to HPH at 70 and 90 °C, in different cycles (3 to 5 cycles) and pressures (500–1500 bar), resulting in an oil/water nanoemulsion. It is important to highlight that due to the reduction of the viscosity of the internal phase, the higher the temperature, the smaller the PS, always paying attention that the temperature control must be compatible with the stability of the active compound. Increasing the homogenization pressure or the number of cycles can result in a problem known as over-processing, which causes an increase in PS due to the recoalescence of the new droplets formed [19,100,101].

Cold HPH is an alternative to the addition of bioactive compounds or heat-sensitive ingredients. In this method, the bioactive compound is dispersed in the molten lipid phase with subsequent rapid cooling in liquid nitrogen or dry ice. The frozen mixture formed by the lipid phase and the bioactive compound is then milled resulting in particles with dimensions between 50–100 μm. During the milling process, it is necessary to ensure that the temperature does not exceed the MP of the lipid with the lowest MP. These micrometric particles are then dispersed in a cold surfactant solution by simple agitation and subjected to HPH to form nanometric-sized particles [99,102]. Cold HPH reduces the thermal degradation of the bioactive compound. In addition, the retention of the bioactive compound is improved, and the high cooling rate favors its uniform distribution within the lipid matrix. Finally, the crystallization process is controllable and rapid cooling can lead to the formation of the desired crystal structure [79,103].

The physicochemical characteristics of LNs obtained by HPH are affected by a set of parameters, which include solubility of the encapsulated component, polymorphism of the lipid matrix, nature and concentration of the lipid phase and emulsifiers, process temperature, shear force, and number of homogenization cycles [47]. Several studies have been reported to obtain LNs using this technique, with favorable results regarding physical properties and stability (Table 2) [15,30,104].

#### 4.1.2. Microfluidization

The principle of this method is similar to HPH, in that it involves high pressure (up to 30,000 psi) to promote the passage of the emulsion through the microfluidizer channels, to facilitate the breakup of the droplet. However, the design of the channels through which the emulsion circulates is different. While in HPH, the emulsion circulates through only one channel; in this method there is a division of the emulsion that flows in a channel into two streams, each passing through a thin channel and then directed to an interaction chamber in which destructive forces are applied to break up the droplet. As in the HPH technique, the size of the droplet tends to decrease with increasing pressure and the number of cycles. This process results in a combination of high pressure, high velocity, vibrations, pressure drop, shear rate, and hydrodynamic cavitation [105]. However, the viscosity of the pre-emulsion must be in a range that facilitates the passage through the equipment and subsequent breakup of the droplets on a nanometric scale [105,106]. The disadvantage of this technique is susceptibility to over-processing, resulting in reduced emulsion stability, requiring optimization of processing conditions [105]. Among the advantages of microfluidization, we can mention the ease in developing LNs with PS smaller than 136 nm, uniformity, and greater process reproducibility [107,108].

Microfluidic devices used in the production of LNs can be categorized into two main types: (i) a chip is designed to allow controlled mixing of organic and aqueous solvents; (ii) the organic solvent is delivered through a central channel surrounded by several external channels that facilitate its rapid dilution in the output aqueous solution [109].

Microfluidization in food processing offers several advantages, including fast processing time, low thermal impact, minimal nutritional loss, high flexibility for increase, and more uniform droplet size distribution. Nanoemulsions obtained by high-pressure microfluidization present characteristics such as reduced droplet diameter, high physical stability, high LC, and optical transparency. These properties favor their application as delivery systems, increasing their efficiency [81,105,110]. The bioactive compounds curcumin and β-carotene have been evaluated for co-encapsulation in a Pickering emulsion stabilized by nanoparticles using microfluidization. The lower pressure (≤100 MPa) provided by microfluidics stimulated lipolysis and increased the bioaccessibility of the nutraceuticals, with an effective increase of the surface area, allowing more nanoparticles to be accommodated at the interface. However, in the case of curcumin, the pressure resulted in reduced encapsulation due to thermal degradation [81].

#### 4.1.3. Ultrasonic Homogenization

In ultrasonic homogenization, the lipid phase is initially heated at 5–10 °C above its MP. Initially, the pre-emulsion is formed where the lipid phase is then dispersed in an aqueous phase, heated, and surfactant is added, under high-speed agitation. This emulsion is subjected to the sonication process that reduces the emulsion droplet size. Gradual cooling of the hot emulsion below the lipid crystallization temperature produces LNs. Ultrasound uses high-intensity ultrasonic waves (20–50 Hz) that generate intense shear and pressure gradients, breaking the droplets mainly through cavitation and turbulent effects [71,111]. Sonication at high temperatures facilitates the dispersion of the solid lipid in the aqueous phase, while the combination of parameters such as time and intensity affect the obtaining of smaller particles [112]. Osanlou et al. (2022) observed nanomaterials with diameters smaller than 150 nm, but LNs for transporting zeaxanthin with different diameters when elaborated with high shear force and ultrasound [37].

The ultrasound method offers the advantage of producing LNs with a small PS (30–180 nm), greater stability, better EE, and low shear stress, in addition to high performance and lower environmental impact. However, certain limitations must be considered, such as the potential for metal contamination due to metal shielding, lower EE, and the high energy consumption of the process. During sonication, interaction with metallic components of the equipment may lead to the release of metal ions, affecting the purity of the final product [112,113].

### 4.2. Low-Energy Methods

#### 4.2.1. Microemulsion

In this method, the initial steps consist of melting and mixing the lipids together with the bioactive compound. To form microemulsion, the lipid phase is added to the aqueous phase, at the same temperature and with the addition of surfactant and co-surfactant. This microemulsion is then dispersed in cold water (2–10 °C) to allow rapid recrystallization of lipid phase droplets and formation of LNs. Dilution factors range between 1:25 and 1:50 (Figure 4a) [114,115,116]. This is a reproducible method and does not use solvents, making it suitable for encapsulation of thermolabile compounds. The ultrafiltration or lyophilization techniques are normally used to remove excess water. The surfactant promotes the spontaneous self-assembly of the hydrophobic or hydrophilic parts in a solvent, forming micelles, bilamellar phases, and reverse micelles. The main advantage of this method is its speed and capability of solubilizing lipophilic components. However, it has the disadvantage of using high concentrations of surfactants and co-surfactants. Water removal by ultrafiltration or lyophilization is difficult and time-consuming due to the small size of the nanoparticles and may modify the physical characteristics of the nanoparticles [113,117,118]. Microemulsion has been applied in several systems for SLNs and NLCs formulations, resulting in loaded SLNs with small PS, excellent physical stability, and controlled drug release [119,120].

#### 4.2.2. Spontaneous Emulsification

An emulsion or nanoemulsion is formed spontaneously when two liquids are mixed. In practice, spontaneous emulsification can be performed in several ways: by varying the composition of the two phases; by changing the environmental conditions (e.g., temperature, pH, and ionic strength); and/or the conditions for homogenizing the emulsion phases (e.g., order of addition of components, stirring speed, addition rate) (Figure 4b). A series of physicochemical mechanisms have been proposed to explain spontaneous emulsification. The mechanism that best elucidates this emulsification is the action of the emulsifying agent, which is partially miscible in the two phases of the emulsion (oil and water). When these two phases are placed in contact, part of this agent migrates from one phase to the other, promoting an increase in the oil/water interface, interfacial turbulence, and the spontaneous formation of droplets. Therefore, the main objective of this method is to change the conditions so that the emulsifier can promote the formation of droplets on a nanometric scale [71].

Spontaneous emulsification is of increasing interest, particularly in nanostructured bioactive delivery systems. This is because spontaneous emulsification is driven by diffusion followed by nucleation and growth. In this case, low interfacial tension plays a secondary role. However, emulsification processes that involve the same phase transitions in surfactant systems can result in emulsions with varying size. Thus, the success of the application usually depends on the careful selection of materials to obtain the desired emulsion properties [121,122].

#### 4.2.3. Solvent Diffusion

In the solvent diffusion, partially water-miscible organic solvents (benzyl alcohol, butyl lactate, isobutyric acid, isovaleric acid, and tetrahydrofuran) are initially saturated with water to ensure initial thermodynamic equilibrium. Subsequently, the lipids and the bioactive compound are dissolved in the water-saturated solvent. This mixture is emulsified in an aqueous emulsifier solution saturated with solvent by a mixer to form an oil-in-water (O/W) emulsion. The O/W emulsion is gradually added to the aqueous phase under continuous stirring, facilitating the removal of the organic solvent by diffusion and promoting the solidification of the dispersed phase and consequent formation of LNs. To optimize the process, emulsion/water ratios generally ranging from 1:5 or 1:10 are used. The solvent can be removed by ultrafiltration or lyophilization [15,123]. Nanoparticles with PS below 100 nm can be obtained, in which surfactants are important for optimizing the size. However, there are disadvantages, such as the need to purify and concentrate the LNs’ dispersion and the permeation of the bioactive compounds [124].

#### 4.2.4. Solvent Injection

**Figure 5:**
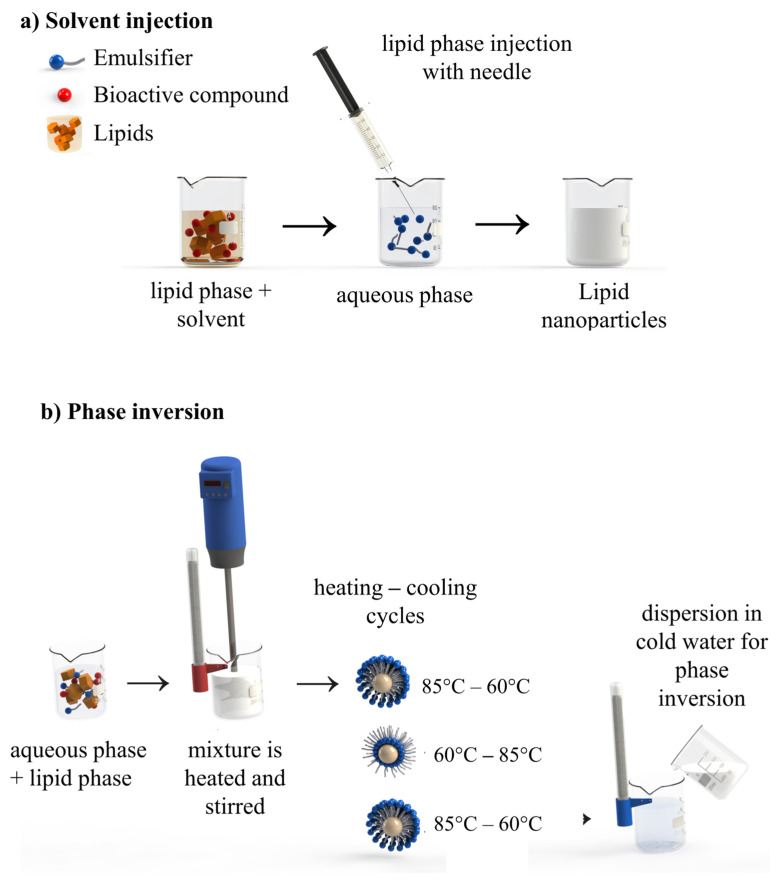
Low-energy methods to produce lipid nanoparticles. (**a**) Solvent injection and (**b**) phase inversion.

The principle is similar to the solvent diffusion method. In the solvent injection method, lipids are dissolved in a water-miscible solvent (acetone, isopropanol, and methanol) or water-miscible solvent mixture rapidly with an injection needle into an aqueous surfactant (Figure 5a). In the solvent injection, the PS of LNs can be influenced and controlled by varying the process parameters, such as type of injected solvent volume, concentration, lipid concentration in the solvent phase, and viscosity of the aqueous phase [125,126]. In this method, the diffusion of the solvent from lipid-solvent droplets into water reduces their size and increases the lipid concentration. Subsequently, the diffusion of the pure solvent from the droplets further decreases their size [127]. This method has the advantages of speed process, easiness of handling, and application in laboratories without the need for expensive equipment [128].

#### 4.2.5. Phase Inversion

Phase inversion is a recent, economical, and solvent-free approach to form LNs. This method is based on the inversion of emulsion phases, for example, from an O/W emulsion to obtain a water-in-oil (W/O) emulsion (and vice versa), through the modification of some condition such as temperature or the composition of the emulsion phases. The change in temperature modifies physicochemical properties of non-ionic surfactant agents, such as relative solubility and molecular geometry, promoting phase inversion. Phase inversion can also occur by modifying the composition of the emulsion phases, such as the addition of salt, for example, which promotes the change in the optimal curve of the surfactant agent and thus the inversion of the phases [71]. In this process, all ingredients (lipids, emulsifier, and water) are mixed in optimized proportions. The mixture is stirred and heated until it reaches 85 °C above room temperature. Then, cooling and heating cycles are applied to the system to reach the phase inversion zone (85–60 °C; 60–85 °C; 85–60 °C). In the second step, this mixture is diluted in cold water (0 °C) to break the system and promote phase inversion (Figure 5b). This rapid addition of cold water forms transparent dispersions with sizes < 25 nm. Slow magnetic stirring for 5 min prevents particle aggregation [129]. Nanoparticles are formed with each phase inversion. The main limitation of this method is that only bioactive compounds that are resistant to high temperatures can be incorporated. In addition, it is necessary that the surfactants do not have their physicochemical properties affected by temperature variation.

## 5. Characterization Methods

The physicochemical properties and functional performance of LNs, including stability, optical properties, delivery rate, and metabolic fate, are influenced by their inherent characteristics (size, formulation, and thermal properties, among others) [130]. Characterizing LNs presents significant challenges due to their small size and the complexity of the systems involved [131]. In this section, the most used methods for characterizing LNs are presented.

### 5.1. Particle Size (PS) and Polydispersity Index (PDI)

PS is one of the most important properties for characterizing LNs. This determination can confirm whether the desired dimensions were obtained using specific formulations and processes and, most importantly, whether these dimensions are maintained during storage or subsequent processing. Furthermore, PS can significantly affect the interaction of LNs in different biological media. This property can be correlated with the composition of the solid and liquid lipid used to produce LNs; with its physicochemical stability; and allows obtaining complementary information about LNs morphology [132]. Controlling LNs dimensions is essential, since this parameter influences their physicochemical properties, functionality, and application potential [29].

Since LNs have dimensions ranging from 10 to 1000 nm, PS is commonly assessed using the dynamic light scattering (DLS) technique, which measures the rate of the intensity fluctuations in scattered light due to the Brownian motion of particles in suspension, using this to generate the PS distribution. The frequency of intensity fluctuations is influenced by the rate at which particles move, which in turn depends on their size. Smaller particles, moving more quickly than larger ones, exhibit faster intensity fluctuations. The sample is analyzed by measuring the intensity fluctuations in the scattered light at a specific scattering angle. These fluctuations are then used to generate the autocorrelation function, where the decay of the curve is proportional to the particle diffusion coefficient. The particle hydrodynamic diameter is subsequently calculated trough the Stokes–Einstein equation and the PS distribution is estimated through a mathematical model [133,134].

In addition to evaluating PS, DLS measurements provide information about the LNs’ homogeneity through the PDI, which represents an estimate of the size distribution width. This index significantly impacts the physical stability of LNs and should be minimized to ensure long-term stability. PDI values between 0.1 and 0.25 indicate a narrow size distribution, suggesting that LNs have similar PS. In contrast, PDI values greater than 0.5 signify a broad distribution and potential instability in the system, which may lead to LN aggregation [18,132]. Prior to the determination of PS and PDI, LN dispersions must be diluted with ultrapure water (from 1:10 to 1:100) to obtain an adequate dispersion intensity (particle count between 100 and 1000 s^−^^1^). This condition occurs when particle–particle interactions are negligible, which is essential for accurately determining the particle size using the Stokes–Einstein equation [111,135]. In the case of samples collected during in vitro digestibility assays, dilution with ultrapure water must be performed at the pH corresponding to each phase of digestion (for example, pH 3.0 for the stomach phase). After dilution, the samples are placed in specific cuvettes for measurement.

### 5.2. Zeta Potential (ZP)

Since the surface potential of particles cannot be measured directly, the ZP is typically used as a characteristic parameter to represent the charge of LNs [136]. ZP is mainly used to provide information on the stability of formulations during storage, allowing important formulation adjustments [132]. The ZP is an indirect measure of the electric potential at the hydrodynamic shear surface surrounding colloidal particles. It represents the distance from the particle surface within which counterions remain strongly bound when the particles move under the influence of an electric field [71].

Colloidal particles typically carry a surface charge, which results from the presence of ionized groups or the adsorption of ions from the surrounding dispersion medium. The magnitude and distribution of this surface charge and the resulting electric field around the particles are crucial factors in determining the electrostatic repulsion between LNs and, consequently, their electrostatic stability [15,137]. ZP values around ±30 mV are considered a good indication of system stability due to electrical repulsion, suggesting a lower tendency to coalescence or flocculation. Positively charged particles typically tend to adhere to negatively charged surfaces or components, and vice versa, due to electrostatic attraction [4,35].

ZP can be determined using analytical instruments that measure electrophoretic or electroacoustic mobility [71]. The most used equipment for determining ZP in LNs relies on measuring the velocity at which particles move when a well-defined electric field is applied. The sample preparation and dilution are typically carried out as described in Section 5.1. The diluted sample is placed in a measuring cell containing a pair of electrodes. The electric field is then applied, causing the charged particles to migrate toward the oppositely charged electrode. The speed at which they move is influenced by the magnitude of their charge and the viscosity of the surrounding medium. Particle movement in the measuring cell can be assessed by several techniques, with laser-light scattering being the most common [138]. The measured electrophoretic mobility is converted into ZP using Henry’s equation, with approximations based on either Huckel’s or Smoluchowski’s models [139]. A method for preparing ZP samples must closely replicate the original medium, particularly in terms of pH, total ionic concentration, and the concentration of any additives that may be present. In general, for ZP measurement, samples are diluted in ultrapure water, adjusting the conductivity (50 μS/cm) with NaCl or KCl solution (0.1% m/v) [111].

### 5.3. Crystallinity and Polymorphism

The degree of crystallinity and polymorphic modifications of lipids are crucial factors in the development of LNs, as these properties are closely linked to the ability to encapsulate compounds and regulate their release rates [31,36]. Moreover, factors such as the type, concentration, and composition of TAGs in the lipid phase, the type and concentration of emulsifiers, and the entrapment of bioactive compounds can significantly influence the polymorphic behavior of LNs.

The thermodynamic stability of LNs and the extent of lipid packing increase, while the incorporation of compounds decreases, in the following sequence: α polymorphic form, β’ polymorphic form, and β polymorphic form [140]. During storage, LNs may undergo rearrangement of their crystal lattices, transitioning to more stable states (α → β’ → β) [141]. These polymorphic transitions and the melting behavior of the lipid phase are essential factors in shaping the crystal structure, in particle morphology, in the release kinetics of bioactive compounds, and in the crystallinity index of LNs. They also indicate the highest temperature at which LNs maintain their solid state [29]. Polymorphic transitions are usually associated with changes in particle morphology, with the structure shifting from spherical (α) to more flattened (β) structures [132].

**Figure 6:**
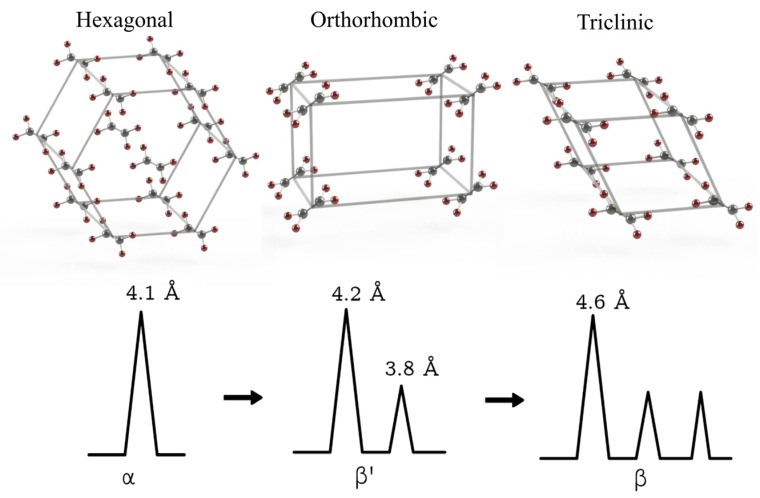
Identification of LN polymorphism by X-ray diffraction. Illustrative diffractograms indicating the characteristic short spacings of the different crystalline phases.

The crystal habit, degree of crystallinity, and polymorphic transitions of LNs are typically evaluated using X-ray diffraction (XRD). The polymorphic forms of LNs can be determined following the AOCS Cj 2–95 method [142], under the specific temperature conditions used for LN stabilization studies. XRD analyses are conducted using a diffractometer with Bragg–Brentano geometry (θ-2θ configuration) and Cu–Kα radiation (λ = 1.54056 Å) at a voltage of 40 kV and a current of 30 mA. Measurements are typically taken in 0.02° steps in 2θ, with a scan time of 2 s and a range from 5° to 40° (2θ scale). Polymorphic forms are identified based on short spacings (SS) characteristic of the crystal structures: the α form shows a single SS at 4.15 Å, the β’ form is identified by two SSs at 3.8 Å and 4.2 Å, and the β form is associated with a high-intensity SS at 4.6 Å and a lower-intensity SSs at 3.8 Å and 4.2 Å, as illustrated in Figure 6 [142].

### 5.4. Thermal Properties and Solid Fat Content (SFC)

Differential scanning calorimetry (DSC) is a commonly employed technique for assessing the physical state of LNs. This method involves measuring enthalpy changes as a sample undergoes a controlled temperature scan [143,144]. DSC analysis is also instrumental in understanding the thermal behavior of the lipid systems used in LN formulation, particularly those composed of lipid mixtures with varying MP [9,17]. Typically, LNs exhibit distinct thermal behavior compared to bulk lipid matrices when evaluated at the macroscale. As a result, it is crucial to perform an initial evaluation of the thermal properties of the lipids used in LN formulation to accurately predict their performance [17,30,36].

In the characterization of LNs, DSC provides valuable information about the physical state and degree of crystallinity by monitoring the thermal behavior during melting. This technique enables the evaluation of key parameters involved in the processes used to produce LNs, as the lipid matrix is heated and subsequently cooled to facilitate LN formation [145]. Furthermore, lipid systems used in the production of LNs must maintain stability at body temperature (37 °C), which is a crucial criterion, especially for those designed to entrap and deliver bioactive compounds. Stability ensures the preservation of the structural integrity of the nanoparticles as they pass through the GIT, which is essential for their effectiveness [17].

The conditions for this analysis generally consider the following parameters: sample mass: ~10 mg; maintenance of isothermal condition: 25 °C for 10 min; melting events evaluated between 25 and 100 °C, at a rate of 10 °C/min [52]. Recrystallization index (RI), which indicates the degree of recrystallization of the lipid phase, can be calculated to assess the physical state of LNs.

The solid fat content (SFC) of LNs at a specific temperature provides important insights into polymorphic transitions, crystallization conditions, and the correlation between solid content and the retention efficiency of encapsulated bioactive compounds. Low-field pulsed Nuclear Magnetic Resonance (NMR) is commonly used to assess the solid content in fats, lipid matrices, and colloidal systems. NMR equipment is capable of quickly analyzing concentrated and optically opaque samples without the need for extensive sample preparation. For this analysis, samples, without dilution, are placed in NMR tubes and kept under isothermal conditions at the stability assessment temperature for a duration of 10 min. The solids content in the LNs is then measured, providing insights into the structural properties of the nanostructures [9,43].

### 5.5. Morphology and Ultrastructure

The morphology of LNs can vary significantly. Spherical particles are particularly advantageous for sustained release and entrapment of bioactive compounds, as they offer extended diffusion routes and reduced interaction with the aqueous medium. Additionally, spherical LNs typically require a lower amount of emulsifier for stabilization due to the minimized specific surface area. In contrast, anisometric particles may offer benefits when encapsulating compounds within the emulsifier layer [134]. It is important to consider the ultrastructure of LNs in studies aimed at producing SLNs and NLCs, as these structural characteristics directly impact the EE and release properties of the bioactive compounds [33].

Several microscopy techniques are available to characterize the morphology and organization of LNs, with optical, electron, and scanning microscopy being the most commonly used. Each method offers distinct advantages and has its own set of limitations, depending on the specific application. Optical microscopy is a straightforward and economical technique, but it is mainly used to observe the positioning, movement, or aggregation of LNs, rather than offering in-depth structural details of individual nanoparticles. Due to the mechanical constraints of optical components and the Brownian motion of LNs, optical light microscopy is unable to accurately resolve particles smaller than 500 nm [138]. In contrast, polarized light optical microscopy can be employed to distinguish optically anisotropic nanoparticles from an isotropic background, allowing for better visualization of structural features [29].

Advanced microscopy techniques are essential for determining the morphology and ultrastructure of LNs. Scanning electron microscopy (SEM), transmission electron microscopy (TEM), and atomic force microscopy (AFM) are commonly employed to obtain detailed information on PS, PDI, morphology, surface characteristics, and internal structure of LNs. Additionally, combined techniques such as Scanning Transmission Electron Microscopy (STEM) and Focused Ion Beam Scanning Electron Microscopy (FIB-SEM) can improve image sensitivity and resolution. For temperature-sensitive materials, image acquisition can be conducted at extremely low temperatures using cryo-SEM, which involves the use of liquid nitrogen to preserve the sample’s structural integrity during imaging. Among the various microscopic techniques, electron microscopy is considered the most effective for characterizing the microstructure and organization of LNs. This is largely due to its ability to detect structural features through an electron beam, offering a resolution of around 1 nm. This high resolution enables the visualization of fine details that are beyond the capabilities of optical microscopy, which is limited by the wavelength of light [29]. Montes et al. (2019) conducted an extensive analysis of these microscopy techniques, discussing critical factors such as sample preparation, operating conditions, advantages, limitations, and practical applications for the characterization of LNs [135].

### 5.6. Entrapment Efficiency (EE) and Load Capacity (LC)

An LN delivery system must exhibit a range of properties to be considered suitable for the entrapment of bioactive compounds, with high EE and LC being among the most important. EE is defined as the ratio of the amount of the bioactive compound encapsulated within the LNs to the total amount of the compound initially solubilized into the lipid phase [28,146]. The EE can be assessed by measuring both the free and encapsulated fractions of the compound of interest [147]. The EE value directly impacts the release properties of the LNs and is influenced by both the components of the formulation and the specific production method employed [29]. Additionally, through EE, the chemical stability of bioactive compounds loaded into LNs during storage is assessed [32]. EE reflects the extent to which these compounds remain entrapped within the LN structures, both during and after storage [28,148].

The LC parameter corresponds to the ratio of the amount of encapsulated compound to the total amount of lipid phase. Factors such as physical structure of LNs, polymorphic form, and the solubility of the bioactive compound in the lipid phase influence the LC [147]. LC reflects the ability of the lipid matrix to carry the desired bioactive compound to the target site for adsorption. LC values range from 0 (low) to 100% (high), and achieving a high LC is advantageous in terms of cost efficiency, as it requires a smaller volume of LNs to deliver the desired compound [29].

The amount of active compound inside and outside the LNs can only be determined after the separation of the nanoparticles from the surrounding environment. Numerous approaches have been proposed to isolate the nanoparticles from the surrounding matrix, including dialysis, filtration, gravity separation, centrifugation, and others. The concentration of the bioactive compound in the LNs can be measured using appropriate analytical tools such as chromatography, electrophoresis, or spectroscopy [138].

### 5.7. Physical Stability

The stability of colloidal systems refers to the ability of an emulsion to retain its properties over time. In other words, the more stable the emulsion, the slower its properties will change [74]. The physical stability of LNs is primarily influenced by two key factors. First, the ability of the LNs’ dispersion to remain homogeneous (i.e., a single phase), which is affected by destabilization phenomena such as flocculation, creaming, sedimentation, and coalescence. Second, the stability of the solid lipid matrix, which determines its resistance to recrystallization over time. Polymorphic transformations may induce recrystallization, leading to the formation of a network of crystallized particles that can result in gel-like behavior [4].

Flocculation is a phenomenon in which two or more particles aggregate while maintaining their individual integrity. This destabilization phenomenon typically occurs due to a lack of sufficient repulsive forces between particles. Additionally, flocculation can be intensified by factors such as agitation or elevated temperature, which increase the frequency of particle collisions within a specific area over a given time period. As a result, flocculation leads to an increase in the viscosity of dispersions, potentially resulting in the formation of a gel-like system [74]. Flocculation often accelerates phase separation driven by gravitational forces, such as sedimentation or creaming. Since LNs generally present a different density than the surrounding liquid (e.g., water), a net gravitational force act on them. If the LNs present a lower density than the surrounding liquid, they tend to rise, a phenomenon known as creaming. Conversely, if the LNs present a higher density, they tend to settle, resulting in sedimentation. Both sedimentation and coalescence are typically reversible processes, as gentle agitation can redisperse the particles, provided that they are not strongly attracted to one another or that coalescence has not occurred [74].

**Figure 7:**
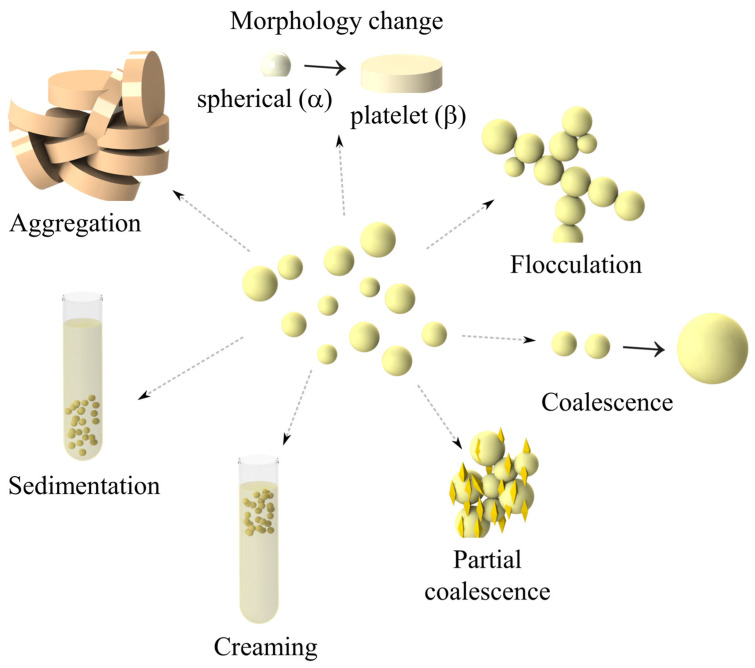
Different physical destabilization phenomena that can occur in lipid nanoparticles formulations.

Destabilization by coalescence occurs when two or more particles merge to form a single, larger particle. This process requires a liquid lipid matrix, which may involve the liquid lipid present in the formulation of LNs. Coalescence can take place during various stages of LN production, such as during evaporation, drying, or even the HPH process [4]. Additionally, partial coalescence can occur in LNs due to the combination of solid and liquid lipids. Partial coalescence happens when droplets formed by partially crystallized lipids interact with the liquid lipid of another droplet, leading to the formation of an aggregate with an irregular shape. The resulting aggregate partially retains the shape of the original droplets because the mechanical resistance from the crystalline lipid network within the droplets prevents complete fusion [149,150]. A schematic representation of the primary physical destabilization phenomena in LNs is shown in Figure 7.

Turbiscan backscattering analysis is a valuable method for evaluating the global stability of emulsions, offering insights into the loss of emulsion stability over time. It complements techniques like PS and ZP analysis by detecting changes that occur before they are visible to the naked eye. The samples are placed in specific tubes and maintained in a stable condition throughout the analysis period, with minimal movement of the tubes. The tube is then inserted into the equipment, which detects changes in the LN’s dispersion. The detection is based on an infrared light source with a wavelength of 880 nm, paired with transmission (T) and backscattering (BS) detectors. Transmission (T) measures the light that passes through the sample, while backscattering (BS) detects the light reflected back from the sample that is not absorbed. The Turbiscan Stability Index (TSI) represents a cumulative sum of either BS or T signals, providing a quantitative assessment of the sample’s global destabilization over time. This index helps identify the destabilization mechanisms, distinguishing between reversible processes (such as creaming or sedimentation, which involve particle migration) and irreversible changes (such as flocculation or coalescence, which involve variations in PS), thereby reflecting the overall stability of the sample [151].

## 6. Conclusions and Future Perspectives

Nanotechnology research highlights the superior functionality of nanoscale release systems compared to conventional encapsulation methods, driving interest in nanocarrier systems known for their high kinetic and thermodynamic stability. Among these, lipid-based nanoparticles are emerging as a promising encapsulation technology. These systems offer distinct advantages, such as varied physicochemical properties, the ability to retain compounds with different solubilities, and enhanced bioavailability due to their composition of organic lipids. The potential of lipid-based nanoparticles is well recognized in the pharmaceutical industry and is increasingly being explored in food science. The potential of LNs in food science can be maximized through the correct and appropriate selection of the lipid matrix, emulsifier type, and production method.

Regarding the production method, the selection of the lipid nanoparticle (LN) production technique depends on the intended application and whether a high-energy or low-energy approach is preferred. Each method has its advantages and disadvantages, which must be evaluated according to the desired purpose, characteristics of the bioactive compound, scalability, and costs. Currently, HPH and microfluidization stand out as promising methods. HPH is widely used due to the commercial availability of homogenizers and ease of scalability. Microfluidization, on the other hand, offers process uniformity and reproducibility, low thermal impact, minimal nutritional loss, and high production speed, making it an attractive alternative for heat-sensitive formulations.

Many studies still focus on high-cost synthetic lipids, which are not always feasible for food applications due to cost and regulatory concerns. A growing trend is the use of edible or commercially available oils and fats in place of synthetic lipids. This shift is motivated by both economic and regulatory factors. Advances in nanoparticle technology for the food industry now prioritize the use of biocompatible lipid fractions, typically derived from natural oils and fats, and emulsifiers from renewable, food-approved sources. Additionally, lipid systems are selected based on their chemical composition and crystallization properties, which are compatible with nanostructure development. This focus on conventional lipid materials from the oil and fat industry presents a strategic opportunity for the widespread application of nanoparticles in food products. In the oil and fat industry, SLNs and NLCs offer significant potential across various applications, including nutraceuticals, functional foods, and advanced delivery systems for flavorings, preservatives, and essential oils. These nanoparticles enhance the stability of processed foods by protecting sensitive compounds from oxidation and degradation. Notable applications include fortified oils, dietary supplements, functional beverages, and the enrichment of lipid-based products, such as margarine fortified with phytosterols. Additionally, LNs are beneficial in coating formulations, where they improve antioxidant and antimicrobial properties. These approaches are further supported by extensive research into the production, characterization, and delivery of bioactive compounds, reflecting the growing interest in nanoparticle-based food formulations.

The application of LNs in the food industry is constrained by several limitations, particularly concerning regulatory and safety issues. Due to their PS, LNs have the potential to cause adverse effects such as gastrointestinal disruption, genomic changes, tissue accumulation, DNA damage, cytotoxicity, and alterations to the gut microbiota [152]. Moreover, concerns related to emulsifiers and the possibility of immunological or toxic responses require thorough evaluation [7]. Regulatory agencies, including the FDA and EFSA, provide safety guidelines for the use of LNs in food products. Consequently, conducting risk assessments and toxicity studies is critical to understanding their health impacts and should be incorporated into the development and application of LNs.

## Data Availability

No new data were created or analyzed in this study. Data sharing is not applicable to this article.
